# Racial disparities in mortality after severe traumatic brain injury in childhood: mediators identified by Oaxaca-Blinder decomposition of trauma registry data

**DOI:** 10.1186/s40621-020-00295-6

**Published:** 2021-01-11

**Authors:** Joseph Piatt

**Affiliations:** 1 Division of Neurosurgery, Nemours / A I duPont Hospital for Children, 1600 Rockland Road, Wilmington, DE 19803 USA; 2 Departments of Neurological Surgery and Pediatrics, Sidney Kimmel Medical College, Thomas Jefferson University, Philadelphia, PA USA

**Keywords:** Adolescents, Children, Decomposition, Infants, Racial disparities, Traumatic brain injury

## Abstract

**Background:**

In the United States social disparities in health outcomes are found wherever they are sought, and they have been documented extensively in trauma care. Because social factors cannot cause a trauma outcome directly, there must exist mediating causal factors related to the nature and severity of the injury, the robustness of the victim, access to care, or processes of care. An understanding these mediators is the point of departure for addressing inequities in outcomes.

**Findings:**

Data were extracted from the registry of the Trauma Quality Improvement Program of the American College of Surgeons for 2007 through 2010. Inclusion criteria were age less than 19 years and head Abbreviated Injury Scale score of 4, 5, or 6. An Oaxaca-Blinder decomposition was undertaken to analyze the relative contributions of a large set of covariates to the difference in mortality rates between Black and White children. Covariates were aggregated into the following categories: “Severity,” “Structure and Process,” “Mechanism,” “Demographics,” and “Insurance.” Eligible for analysis were 7273 White children and 2320 Black children. There were 1661 deaths (17.3%) The raw mortality rates were 15.6 and 22.8% for White and Black children, respectively. Factors categorized as “Severity” accounted for 95% of the mortality difference, “Mechanism” accounted for 13%, “Insurance” accounted for 5%, and “Demographics” accounted for 2%. The contribution of “Structure and Process” did not attain statistical significance.

**Conclusions:**

Severity of injury accounts for most of the disparity between Black and White children in traumatic brain injury mortality rates. Mechanism, insurance status, and gender make a small contributions. Because insurance status like other social factors cannot directly affect trauma survival, what mediates its contribution requires further study.

## Background

In the United States racial disparities in health outcomes can be demonstrated for a great variety of conditions affecting patients of all ages. Traumatic brain injury (TBI) in childhood is no exception. A recent analysis of data from the Trauma Quality Improvement Program (TQIP) of the American College of Surgeons (ACS) has confirmed many previous observations of racial disparities in TBI mortality and presented evidence that factors captured in the TQIP dataset accounted for those disparities (Piatt 2020). Specifically, this study took Black race and White race as ‘treatments’ and used stabilized inverse probability weighting to perform a non-inferiority propensity score analysis. The a priori inferiority margin was 10%. The weighted relative mortality risk for Black children compared to White children was 0.9986 with a confidence interval upper limit of 1.0865. That is, after statistical adjustments for many covariates reflecting severity of injury, mechanisms of injury, insurance status, demographic factors, and processes of care, Black children experienced a risk of mortality no greater than White children. This conclusion raises 2 immediate questions: Which factors account for the disparities? And can they be modified to promote health equity?

The current work addresses these questions with a methodology borrowed from economics and public policy, the Oaxaca-Blinder decomposition (OBD) (Oaxaca 1973; Blinder 1973). It uses the OBD to dissect the contributions of several distinct classes of covariates to the disparity between TBI mortality rates for Black and White children in the TQIP dataset.

(Fairlie 2020)

## Methods

OBD begins with a statistical model of the outcome of interest, in this case mortality after childhood TBI, and asks the counterfactual question, “How much disparity would remain if the disadvantaged group had the same values for the model covariates as the advantaged group?” The mathematics of the OBD is readily understood in the case of a linear model such as least-squares regression. First, models are developed for both groups:

where Ypred_i_ is the predicted outcome, β is the vector of model parameter estimates, and X_i_ is the vector of covariates for the i^th^ member of the advantaged (A) or disadvantaged (D) groups. In least-squares regression the average of the predicted outcomes is equal to the average of the observed outcomes, so

$$ {\mathrm{Y}}_{\mathrm{A}}\mathrm{mean}={\upbeta}_{\mathrm{A}}'{\mathrm{X}}_{\mathrm{A}}\mathrm{mean}\kern0.5em \mathrm{and}\kern0.5em {\mathrm{Y}}_{\mathrm{D}}\mathrm{mean}={\upbeta}_{\mathrm{D}}'{\mathrm{X}}_{\mathrm{D}}\mathrm{mean} $$

The outcome disparity or difference can be written

$$ {\mathrm{Y}}_{\mathrm{A}}\mathrm{mean}-{\mathrm{Y}}_{\mathrm{D}}\mathrm{mean}={\upbeta}_{\mathrm{A}}'{\mathrm{X}}_{\mathrm{A}}\mathrm{mean}-{\upbeta}_{\mathrm{D}}'{\mathrm{X}}_{\mathrm{D}}\mathrm{mean} $$

Adding and subtracting β_D_’X_A_mean from the right-hand side of this equation and rearranging terms yields

$$ {\mathrm{Y}}_{\mathrm{A}}\mathrm{mean}-{\mathrm{Y}}_{\mathrm{D}}\mathrm{mean}=\left({\beta}_{\mathrm{A}}-{\beta}_{\mathrm{D}}\right)'{\mathrm{X}}_{\mathrm{A}}\mathrm{mean}-{\beta}^{\mathrm{D}{\prime}}\left({\mathrm{X}}_{\mathrm{D}}\mathrm{mean}-{\mathrm{X}}_{\mathrm{A}}\mathrm{mean}\right). $$

The second term on the right-hand side represents the portion of the disparity that would disappear if the means of the covariates of the disadvantaged group could be made equal to the means of the covariates of the advantaged group. The first term represents the irreducible disparity attributable to the differences in the model parameters, that is, the difference in the effects of the covariates on the outcomes in the 2 groups. The implementation of OBD for non-linear models, such as logistic regression, relies heavily on iteration, but the interpretation is analogous (Fairlie 2020).

This method is applied to a retrospective cohort study of mortality after traumatic brain injury (TBI) undertaken using data from the TQIP for the years 2014 through 2017 (Committee on Trauma, American College of Surgeons. Trauma Quality Programs (TQP) Participant User Files (PUF) Version 1.0 Chicago, Illinois, 2019). The TQIP is a voluntary program open to trauma centers verified by the ACS. Data is abstracted by trained registrars at the time of discharge. Participating institutions receive reports comparing their quality benchmark performance with the performances of other participating institutions. In this data set race is defined based on parental statements at the time of hospital registration. There were 2 fields for race to permit parents to designate mixed race, and there was 1 field for ethnicity. Observations coded as Hispanic were excluded. Then if ‘BLACK’ appeared in either race field, the observation was categorized as Black. White observations had ‘WHITE’ in the first field and a blank in the second. Mortality rates for Black and White children were compared, because these 2 social categories consistently exhibit the largest outcome differences. The difference between raw mortality rates for Hispanic children and White children in this data set were small and did not attain statistical significance (data not shown).

Organization of data has been described in detail in a recent companion publication (Piatt 2020). The inclusion criterion was a head Abbreviated Injury Scale (AIS) score of 4, 5, or 6 (Gennarelli and Wodzin 2006). This criterion was utilized for 2 reasons. First, many cases did not have valid Glasgow Coma Scale (GCS) scores, and second, this criterion yielded a sample with mortality rates sufficiently large for analysis. Observations coded for discharge from the emergency department (ED) and for transfer to another short-term acute-care facility were excluded. Cases older than 18 years of age were excluded. There were no observations with age = 0, and for a substantial number of observations age was missing. Weight was not so sparse at age, so observations with weight < 9 kg were assigned age = 0. The remaining observations with missing age and weight < 16 kg (the 99th %ile for observations originally coded for age = 1) were coded age = 1. Age was then categorized as ‘infant’ (0 years), ‘toddler’ (2 and 3 years), ‘child’ (4 through 13 years), and ‘teen’ (14 through 18 years). Insurance status was designated ‘commercial’ or ‘non-commercial.’ In the latter category were observations coded as Medicaid, Medicare, ‘self-pay,’ or ‘not billed.’ Cases coded as ‘other government’ were included in the ‘commercial’ category, as they were likely to be military dependents or beneficiaries of other government employees. All quantitative covariates were reduced to categories, as for age so also for Injury Severity Scale (ISS) score, systolic blood pressure in the emergency department (ED), emergency medical services (EMS) minutes, numbers of protective devices, blood product administration, and numbers of diagnoses. Missing data were designated as a separate category or folded into a category with a similar mortality rate.

Covariates for the OBD were selected by manual backward stepwise logistic regression modeling mortality. Covariates associated with mortality at the *p* < 0.10 level were retained. The final model had a c-statistic of 0.964 and a Brier score of 0.0493. Because the they were numerous, and because of high degrees of collinearity among them, the covariates were aggregated in the following categories: ‘Severity’ included AIS scores, GCS motor scores, ISS scores, numbers of diagnoses, systolic blood pressure in the ED, EMS minutes from dispatch to ED arrival, ED disposition, and ventilator days. “Mechanism” included penetrating injury and utilization of protective devices. Insurance status was its own category, as there were no other TQIP metrics of socioeconomic status. “Demographics” was limited to age, as gender did not retain significance in the model. “Structure and Process” included bed size and self-transport. EMS minutes was considered a severity factor because, conditioned on other covariates reflecting severity, shorter EMS times were still associated with greater risk of mortality. Apparently proximity to the hospital conveys additional information about injury severity not reflected in more conventional covariates.

Data analysis was performed with SAS 9.4 (SAS Institute, Inc.; Cary, North Carolina). Code for an SAS macro was downloaded from https://people.ucsc.edu/~rfairlie/decomposition/. This macro calculates the risk difference between advantaged and a disadvantaged subjects and estimates absolute and fractional contributions of groups of covariates to this difference with 95% confidence intervals. It does so by repeated comparisons of the Black cohort with an equal number of randomly sampled White children (Fairlie 2020). The order of the covariates in the logistic regression model is varied randomly with each repetition as well. 5000 iterations of the algorithm were performed. The results can be interpreted as the counterfactual difference in mortality that might be expected if the observed covariates of the Black children in each category were swapped randomly for the covariates of White children. The sum of the counterfactual category differences may be less than the raw difference if there is an irreducible component.

The ACS requires the following acknowledgement: “The content reproduced from the TQP PUF remains the full and exclusive copyrighted property of the American College of Surgeons. The American College of Surgeons is not responsible for any claims arising from works based on the original data, text, tables, or figures.” No content from the TQP PUF has been reproduced in this manuscript.

This project was judged not to be human subjects research by the Office of Human Research of Thomas Jefferson University.

## Results

As reported previously there were 9772 cases meeting the study definition of TBI, 6520 males (66.7%) and 3252 females (33.3%). Survival data were missing for 179 cases. Among cases with survival data there were 7273 White children and 2320 Black children. There were 1661 deaths (17.3%) The raw mortality rates were 15.56 and 22.80% for White and Black children, respectively for a mortality difference of 7.24%.

The associations with race of the covariates retained in the mortality model are depicted in Table 1. Motor GCS, head AIS, and ISS scores were worse for Black children. EMS minutes were shorter for Black children. Penetrating injuries were infrequent but were much more common among Black children. Non-commercial insurance was much more prevalent among Black children. Many other associations were less striking but attained significance because of large numbers of observations.

**Table 1:** Univariate associations with race of covariates from the mortality model

Covariate	Category	Black	White	*p* - value
Motor GCS score				< 0.0001
	6	976 (42%)	3891 (54%)	
	5	219 (9%)	537 (7%)	
	4	166 (7%)	362 (5%)	
	3	56 (2%)	154 (2%)	
	2	51 (2%)	103 (1%)	
	1	579 (25%)	1233 (17%)	
	missing	273 (12%)	993 (14%)	
Head AIS score				< 0.0001
	4	1301 (56%)	4416 (61%)	
	5	989 (43%)	2830 (39%)	
	6	27 (0%)	30 (1%)	
ISS score				< 0.0001
	1 to 17	657 (28%)	2429 (33%)	
	18 to 29	1062 (46%)	3176 (44%)	
	> 29	601 (26%)	1668 (23%)	
Number of diagnoses				0.0506
	1 to 4	808 (35%)	2658 (37%)	
	5 to 14	994 (43%)	2908 (40%)	
	> 14	518 (22%)	1707 (24%)	
Systolic blood pressure in ED				< 0.0001
	low	166 (7%)	289 (4%)	
	normal	1887 (81%)	6295 (87%)	
	high	132 (6%)	298 (4%)	
	missing	135 (6%)	391 (5%)	
EMS minutes				< 0.0001
	< 40	674 (29%)	778 (11%)	
	40 to 127	574 (25%)	2626 (36%)	
	> 128	266 (11%)	1254 (17%)	
	missing	806 (35%)	2615 (36%)	
ED disposition				< 0.0001
	dead	84 (4%)	128 (2%)	
	ICU, OR	1840 (79%)	5675 (78%)	
	other admission	320(14%)	1152 (16%)	
	missing	76 (3%)	318 (4%)	
Penetrating injury				< 0.0001
	no	243 (10%)	227 (3%)	
	yes	2077 (90%)	7046 (97%)	
Protective devices				0.0002
	none	1894 (82%)	5671 (78%)	
	some	426 (18%)	1602 (22%)	
insurance				< 0.0001
	commercial	663 (29%)	4101 (56%)	
	non-commercial	1611 (69%)	3052 (42%)	
	missing	46 (2%)	120 (2%)	
Age group				< 0.0001
	infant	505(22%)	1313 (18%)	
	toddler	228 (10%)	633 (9%)	
	child	694 (30%)	2305 (32%)	
	teen	717 (31%)	2618 (36%)	
	missing	176 (8%)	404 (6%)	
Hospital bed size				< 0.0001
	< 200	157 (7%)	533 (7%)	
	201–400	806 (35%)	2319 (32%)	
	401–600	512 (22%)	2164 (30%)	
	> 600	845 (36%)	2257 (31%)	
Teaching status				< 0.0001
	university	1800 (78%)	5156 (71%)	
	community	443 (19%)	1756 (24%)	
	non-teaching	64 (3%)	293 (4%)	
	missing	68 (1%)	13 (1%)	
Self-transportation				0.0004
	no	1875 (81%)	5624 (77%)	
	yes	445 (19%)	1649 (23%)	

*AIS* Abbreviated Injury Scale, *ED* emergency department, *EMS* emergency medical services, *GCS* Glasgow Coma Scale, *ICU* intensive care unit, *ISS* injury severity scale, *OR* operating room

The estimated contributions of each of the covariate groups to the overall mortality difference is presented in Table 2. The “Severity” covariates make the largest contribution by far to the overall mortality rate difference. “Mechanism,” “Insurance,” and “Demographics” make successively much smaller contributions, and the confidence interval for “Structure and Process” includes the null.

**Table 2:** Oaxaca-Blinder decomposition of the difference in mortality rates for black and white children after severe traumatic brain injury. The observed mortality rate difference was 7.24%. The sum of the estimated contributions was 8.51%. Covariate groups are defined as follows: “Severity” includes AIS scores, GCS scores, ISS scores, numbers of diagnoses, systolic blood pressure in the ED, EMS minutes, ED disposition, and ventilator days. “Mechanism” includes penetrating injury and utilization of protective devices. “Insurance” is limited to payer – commercial or non-commercial. “Demographics” is limited to age group. “Structure and Process” includes bed size, teaching status, and self-transport

Covariate groups	Mortality rate difference (%)	95% confidence interval mortality rate difference	Fractional contribution
Severity	6.89	6.43 to 7.35	95.2%
Mechanism	0.90	0.67 to 1.13	12.5%
Insurance	0.39	0.14 to 0.65	5.4%
Demographics	0.15	0.01 to 0.28	2.1%
Structure and Process	0.17	−0.08 to 0.42	2.4%
Total	8.51	7.87 to 9.15	117.6%

*AIS* Abbreviated Injury Scale, *ED* emergency department, *EMS* emergency medical services, *GCS* Glasgow Coma Scale, *ICU* intensive care unit, *ISS* injury severity scale, *OR* operating room

## Discussion

Injury severity plainly accounts for most of the mortality disparity between Black and White children suffering severe TBI. Mechanism of injury is closely related conceptually, and it accounts for the next largest estimated contribution. “Insurance” and “Demographics” make smaller but significant contributions. The contribution of “Structure and Process” did not attain statistical significance. With reference to the fundamental OBD paradigm, there appears to be no irreducible component. Notably the sum of the estimated contributions of the groups of covariates is greater than unity, 117% of the observed disparity. This result suggests the existence of unmeasured covariates that actually confer a survival advantage on Black children (Robert Fairlie, PhD, personal communication). That covariates collected in TQIP more than explain the greater mortality of Black children after severe TBI suggests that the potential contributions of unmeasured, speculative factors reflecting the robustness of the victims - such as dietary deficiency, environmental toxicity, gene polymorphisms, and epigenetic effects - appear to be minimal, unless such factors selectively disadvantage White children.

The small but persistent and significant importance of insurance status remains a cipher. Like the effect of race, the effect of insurance status on trauma mortality must be mediated by other factors. The current analysis confirms past work indicating that economic status, as reflected in insurance coverage, has an association with trauma mortality independent of race and possibly more important than it (Haider et al. 2008; Rosen et al. 2009; Salim et al. 2010; Gerry et al. 2016). Trauma data sources that are granular with respect both to clinical and to economic covariates are needed to address this question more conclusively, but none are known to the author.

Review of the substantial literature on racial disparities in trauma outcomes is beyond the scope of this report: Black patients have worse outcomes in all age groups after unselected trauma and after traumatic brain injury in particular. Mechanisms and severities of injury, age distributions, comorbidities, access to care, and processes of care have been recognized to differ among socially defined groups of patients, but so far as the author is aware, multivariate models incorporating such factors have not been “decomposed” to analyze their relative contributions, as in the current report (Marcin et al. 2003; Shafi et al. 2007; Rangel et al. 2008; Wood et al. 2010).

Of particular concern has been treatment of socially less privileged groups at worse performing hospitals (Glance et al. 2013; Haider et al. 2013; Hicks et al. 2015). Glance et al. studied mortality, complications, and failure-to-rescue among adult trauma patients using data from the Pennsylvania Trauma Outcomes Study in Level I and II trauma centers over a 10-year period. In models adjusted for clinical factors and access to care, stable racial outcome disparities were demonstrated over the study period. In models additionally adjusted for hospital performance, racial lost significance as a covariate. Conversely, facility-wise odds of adverse outcomes were strongly associated with the fraction of Black patients in the racial mix. The authors concluded that racial disparities in adult trauma outcomes were entirely attributable to variations in hospital performance. Haider et al. analyzed blunt trauma among adults in the National Trauma Data Bank from 2007 to 2010 (Haider et al. 2013). Using a model predicting mortality based on clinical factors and comparing observed to expected deaths, they designated trauma centers as low- or high-mortality. Black and Hispanic patients clustered at high-mortality centers. After adjustment for clinical factors, minority patients treated at low-mortality centers had lower risk of death. In subsequent work based on the Nationwide Inpatient Sample from 2003 through 2009, this group found that the association of race with hospital performance was age-dependent, holding for younger trauma victims but not for older (Hicks et al. 2015). In the current analysis hospital performance was reflected in the “Structure and Process” group in covariates such as ACS verification level, state verification level, bed size, teaching status, incoming transfer, self-transport, and minutes in the ED. Only bed size and self-transport retained significance in the multivariate mortality model. No contribution of “Structure and Process” was apparent. Unfortunately the current study could not address the question of hospital performance directly, as the 2017 edition of TQIP did not include a facility code that had been present in previous years. Analysis of individual facility performance for the entire data set was not possible. Nevertheless, the findings of the current study and the companion non-inferiority analysis leave little room for a major effect of facility performance (Piatt 2020). As the goal of voluntary participating in TQIP is suppression of performance variation, limited inter-facility variation may explain this negative finding.

The original motivation for development of the OBD and other decomposition methods was public policy. It was to determine whether interventions to make disadvantaged subjects look more like advantaged subjects with respect to a specific covariate or group of covariates can minimize an outcome disparity. In the specific case of childhood TBI, the apparent unimportance of structures and processes of care is perhaps disappointing, as such disparities might be relatively tractable to large-scale quality improvement initiatives or policy interventions. Instead, the current analysis directs attention to severity of injury and, with a lesser degree of certainty, to mechanism of injury. Interventions addressing these factors will require great imagination calibrated to specific social, economic, and behavioral conditions.

### Limitations

The findings of the current study are at some variance with the existing literature on racial disparities in trauma outcomes. Certain limitations must be born in mind. While racial outcome disparities seem to be pervasive in medicine and surgery in the United States, the explanations are likely to be specific to the particular disease entity and the age group under study. Furthermore, the data for the current study were collected at institutions that have committed substantial resources to participate in a highly structured quality improvement program. They may not be generalizable to the experiences of children treated in other settings or to children from other racial or ethnic groups. Further work is needed to assess the contribution of variations in facility performance to outcome disparities in pediatric trauma.

## Conclusions

Severity of injury accounts for most of the disparity in TBI mortality rates between Black and White children in the United States. Mechanism, insurance status, and age make much smaller contributions. Because insurance status cannot directly affect trauma survival, the factors that mediate its contribution require further study.

## Data Availability

With the permission of the American College of Surgeons Committee on Trauma, the author will make study data available in response to reasonable requests.

## Abbreviations

ACS: American College of Surgeons
AIS: Abbreviated Injury Scale
ED: emergency department
EMS: emergency medical services
GCS: Glasgow Coma Scale
ISS: Injury Severity Scale
OBD: Oaxaca Blinder decomposition
PUF: Participant User Files
TQP: Trauma Quality Programs
TQIP: Trauma Quality Improvement Program
TBI: Traumatic brain injury
